# Plant virus particles with various shapes as potential adjuvants

**DOI:** 10.1038/s41598-020-67023-4

**Published:** 2020-06-25

**Authors:** Ekaterina A. Evtushenko, Ekaterina M. Ryabchevskaya, Nikolai A. Nikitin, Joseph G. Atabekov, Olga V. Karpova

**Affiliations:** Department of Virology, Faculty of Biology, Lomonosov Moscow State University, 1-12 Leninskie Gory, Moscow, 119234 Russian Federation

**Keywords:** Adjuvants, Biomaterials - vaccines

## Abstract

Plant viruses are biologically safe for mammals and can be successfully used as a carrier/platform to present foreign epitopes in the course of creating novel putative vaccines. However, there is mounting evidence that plant viruses, their virus-like and structurally modified particles may also have an immunopotentiating effect on antigens not bound with their surface covalently. Here, we present data on the adjuvant properties of plant viruses with various shapes (Tobacco mosaic virus, TMV; Potato virus X, PVX; Cauliflower mosaic virus, CaMV; Bean mild mosaic virus, BMMV) and structurally modified TMV spherical particles (SPs). We have analysed the effectiveness of immune response to individual model antigens (ovalbumin, OVA/hen egg lysozyme, HEL) and to OVA/HEL in compositions with plant viruses/SPs, and have shown that CaMV, TMV and SPs can effectively induce total IgG titers to model antigen. Some intriguing data were obtained when analysing the immune response to the plant viruses/SPs themselves. Strong immunity was induced to CaMV, BMMV and PVX, whereas TMV and SPs stimulated considerably lower self-IgG titers. Our results provide new insights into the immunopotentiating properties of plant viruses and can be useful in devising adjuvants based on plant viruses.

## Introduction

The unique properties of plant viruses, including their ability to self-assemble and biosafety for mammals and humans, make them an exceptionally attractive and versatile tool for biotechnology^1,2^. In recent decades, possible applications of plant virus particles and their virus-like particles (VLP) in vaccine development have been studied intensively^3–5^. Some features of plant virus particles’/VLPs’ structural organisation and size allow them to effectively stimulate the immune system^6,7^. Plant virus particles’ immunostimulatory properties can be explained by the fact that they consist of proteins, which are phylogenetically distant and immunologically alien to the mammalian immune system^8^. However, an organised regular spatial structure is considered to be even more important^3,6,8^. In a number of studies, disassembled coat protein (CP) has been demonstrated to be less immunogenic than virions and VLP^9–11^. Effective uptake by professional antigen-presenting cells (APCs) and transportation to lymph nodes has been shown for TMV, wherein its particulate structure has been a key factor^6,12,13^. Therefore, it is likely that CP polymers of plant viruses and their VLP act as pathogen-assoсiated molecular patterns, which are recognised by innate immune receptors^11^. Thus, plant virus can mediate the uptake of target antigen by APCs and thereby promoting the following induction of humoral and cell immune response to that antigen^6^. Nucleic acid, as a constituent part of a plant virus, and some virus-like particles may also contribute to an immune response by stimulating innate immunity receptors, (in particular, TLR7, TLR8 and TLR9)^14–16^.

In the majority of studies focused on the design of plant virus-based vaccine candidates, plant viruses/VLP are used as a carrier/platform for the multiple presentation of a desired antigen/epitope onto the particle surface as part of a CP. In this case the foreign amino acid sequence is fused to the viral CP and attachment is usually achieved by means of genetic engineering or chemical conjugation^3^. However, there is some evidence that the plant viruses/VLP and the plant virus structurally modified particles can possess adjuvant properties without covalently binding of an antigen/epitope to their surface^17–21^.

Our group has been intensively studying the properties of structurally modified particles formed by the thermal remodelling of helical plant viruses^22–27^. The heating of a rod-shaped tobacco mosaic virus (TMV) leads to the transition of virions into spherical particles (SPs). Adjusting the initial concentration of TMV enables SPs of a given size to be obtained^22,24^. We have shown that the SPs can form complexes with proteins, polymers or even small viral particles by simple adsorption alone^18,20,21,28–30^. One of the most interesting properties of SPs is their ability to enhance the immune response to the antigen adsorbed onto their surface^18,20,21^. Notably, SPs have been reported to improve the protectivity of the inactivated rabies vaccine. This effect has been demonstrated to be comparable with the effect of an incomplete Freund’s adjuvant on the same vaccine^31^. In another work, we have demonstrated that SPs had no toxicological effects on mice, rats and rabbits^32,33^.

Modern vaccinology focuses on searching for effective, safe and low-cost adjuvants^34^. As discussed above, the plant viruses/VLP and SPs could be promising candidates for contributing to the design of novel adjuvants.

Here, we present the first comparative study of the adjuvant properties of plant viruses with different structure, size and shape, and TMV SPs. We used two helical RNA plant viruses, rod-like TMV and filamentous potato virus X (PVX), and two icosahedral viruses, cauliflower mosaic virus (CaMV, DNA genome) and bean mild mosaic virus (BMMV, RNA genome). The dose of plant viral particles (100 μg) was selected according to the previous studies on the immunostimulant properties of plant viruses and SPs^21,35^. Ovalbumin (OVA) and hen egg lysozyme (HEL) were selected as model antigens. These proteins are non-infectious antigens and are widely used in studies of adjuvants and delivery systems^17,36–38^. OVA is also known as a poor immunogen^36,37^. In our research, we decided to administer relatively low doses of antigens (5 μg per dose), for better discrimination of the differences between potential adjuvants, and so that the total protein dose in immunogenic compositions would be clinically acceptable.

## Results

### Potentiation of immune response to the OVA

**Figure 1:**
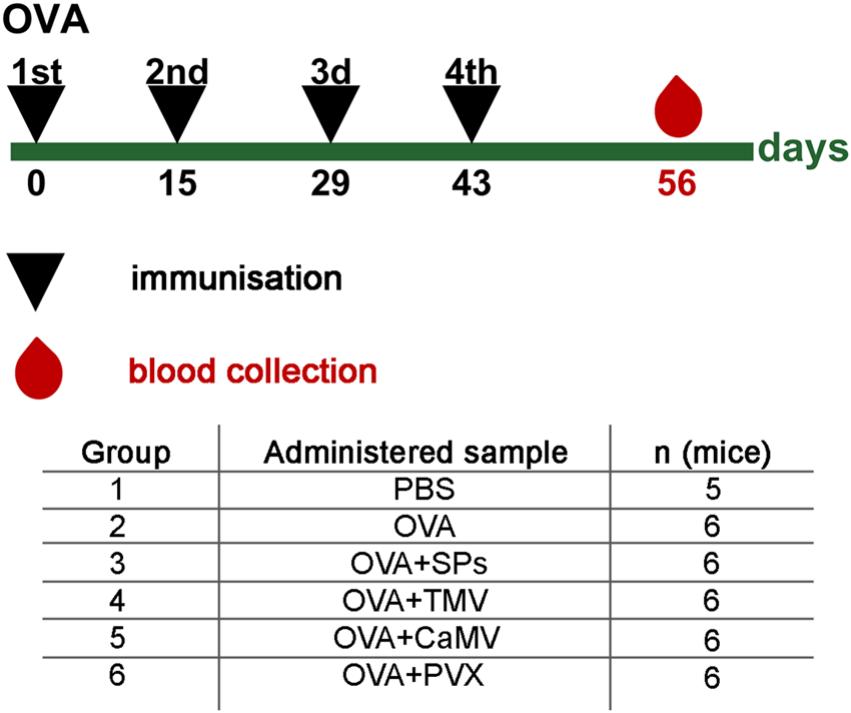
Immunisation schedule and description of animal groups in experiment with OVA. Groups of CD1 mice were immunised s.c. either with 5 μg of OVA or with 5 μg of OVA in composition of 100 μg of plant virus-based adjuvant (SPs/TMV/PVX/CaMV). The control group was immunised with PBS. All administered samples were in PBS in total volume 0.2 ml. OVA, ovalbumin;TMV, Tobacco mosaic virus; CaMV, Cauliflower mosaic virus; PVX, Potato virus X; SPs, spherical particles obtained by thermal remodelling of TMV; n, number of animals.

**Figure 2:**
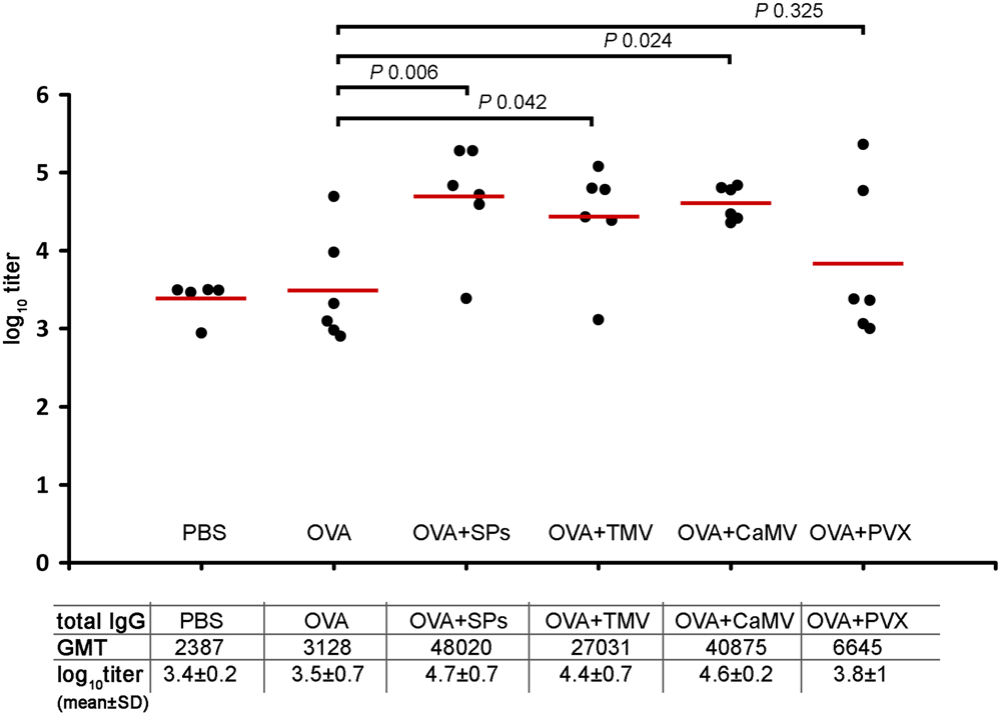
Activation of immune response to OVA by plant viruses/SPs. Groups of mice were immunised s.c. four times (days 0, 15, 29, 43). Blood was collected after the fourth immunisation on the 56^th^ day of the experiment. Sera titers were evaluated using an indirect ELISA with anti-mouse HRP conjugate against IgG (ab6728, Abcam, Cambridge, MA, USA). Concentration of OVA on microplate – 10 μg/ml, ● – anti-OVA serum log_10_titer from each mouse,  – mean. *P* values were calculated using a post hoc Dunn’s multiple-comparison test, which was conducted after a Kruskal-Wallis test. Kruskal-Wallis test *P* value: 0.044. GMT, geometric mean titer; SD, standard deviation.

Mice were inoculated at days 0, 15, 29 and 43, subcutaneously (s.c.), with PBS, OVA (42.7 kDa^39^, pI 4.6–4.9^40^) and OVA-plant virus/SPs compositions (Fig. 1). OVA-virus/SPs compositions were characterised using transmission electron microscopy (TEM) (Supplementary Fig. S1). The mean diameters of SPs and CaMV, measured with ImageJ software (NIH, USA), were 610.6 ± 70.5 and 34.6 ± 2 nm (Supplementary Fig. S1c, g). The mean length of TMV and PVX virions was 315 ± 26.5 and 520.5 ± 39.2 nm (Supplementary Fig. S1e,i), respectively. The TEM analysis of OVA mixtures with SPs, TMV, PVX and CaMV enabled the making of an assumption about the existence of a possible non-covalent-interaction of SPs, TMV and PVX with OVA (Supplementary Fig S1b, d, h), which was not revealed for CaMV (Supplementary Fig S1f). Blood was collected on the 56^th^ day. To determine immune response, an indirect ELISA analysis of mice sera was performed (Fig. 2; Supplementary Table S1). The geometric mean titer (GMT) was calculated for each group. The antibody GMT to OVA considerably increased (by at least 9 times) in groups 3, 4 and 5 as compared with the antibody GMT induced by OVA alone (group 2), while PVX (group 6) did not show any effective stimulation of anti-OVA IgG titers as compared with free OVA. The analysis of IgG1, IgG2 and IgG2b isotypes showed that CaMV increased the anti-OVA GMT by 15 (*P* value 0.038), five (*P* value 0.026) and five (*P* value 0.041) times, respectively, in comparison with free OVA (Supplementary Fig. S2). No significant increase in IgG1, IgG2a or IgG2b to OVA was revealed in sera from groups 3, 4 and 6 as compared with group 2. IgG1, IgG2a and IgG2b anti-OVA titers from each mouse are presented in Supplementary Table S2–S4. IgG3 antibodies were not detected in sera pools of any group at a dilution of 1/50 (data not shown).

### Immune response to the adjuvants/platforms in composition with OVA

**Figure 3:**
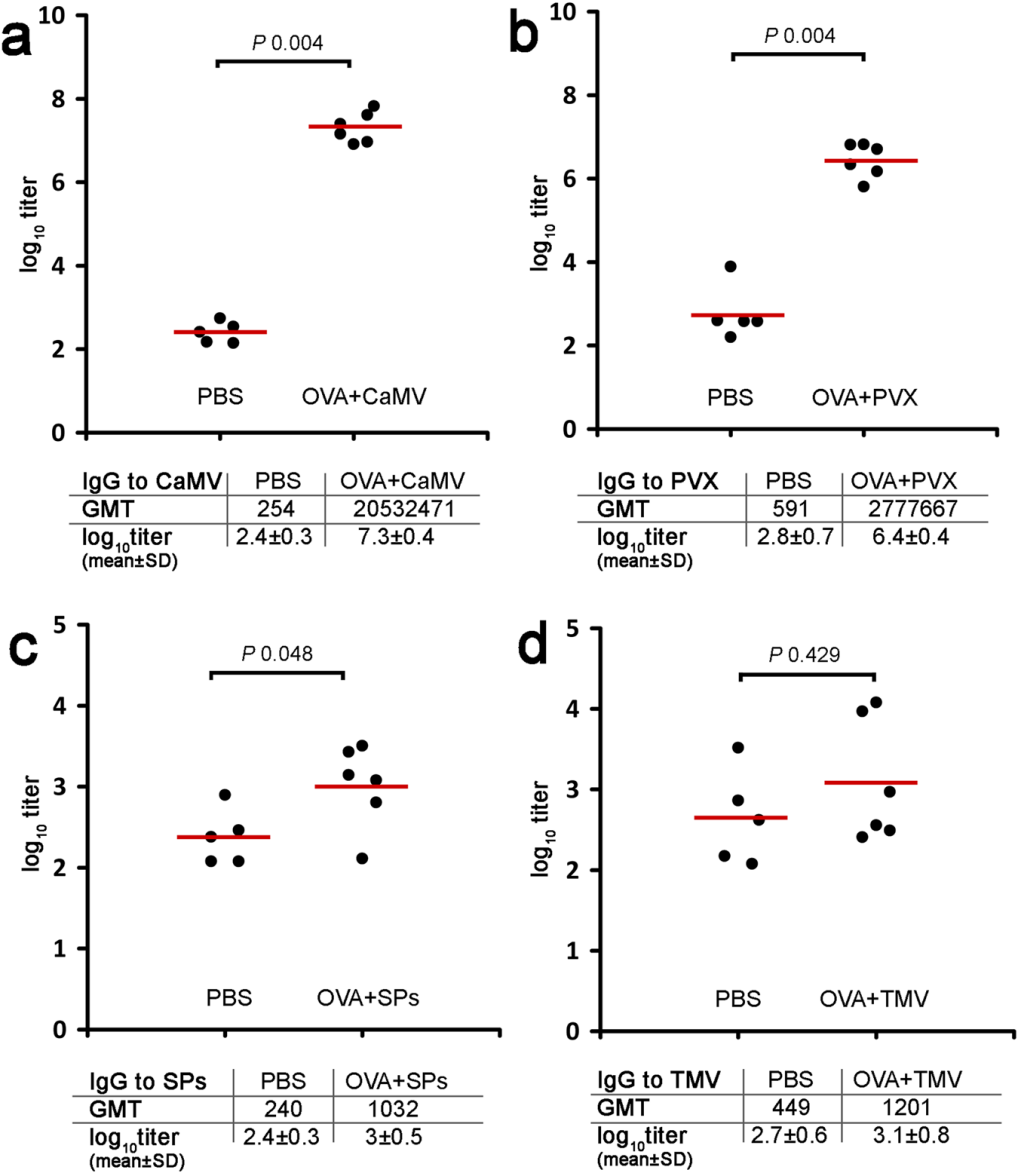
Analysis of total IgG titers to potential adjuvants after immunisation with OVA-plant virus particles compositions. (**a)** total IgG titers to CaMV; (**b)** total IgG titers to PVX; **c**, total IgG titers to SPs; (**d)** total IgG titers to TMV. Groups of mice were immunised s.c. four times (days 0, 15, 29, 43). Blood was collected after the fourth immunisation on the 56^th^ day of the experiment. Sera titers were evaluated using an indirect ELISA with anti-mouse HRP conjugate against IgG (ab6728, Abcam, Cambridge, MA, USA). Concentration of TMV, SPs, PVX on microplate – 10 μg/ml of CaMV – 1 μg/ml, ● – serum log_10_titer from each mouse,  – mean. A Wilcoxon-Mann-Whitney two-tailed test was used to compare the statistical differences among the groups. GMT, geometric mean titer; SD, standard deviation.

To fully describe the adjuvant potential of the used viral particles, it was necessary to evaluate the levels of self-IgG. The analysis of the immune response to the potential adjuvants provided some intriguing data. CaMV and PVX in composition with OVA induced a strong immune response on themselves (Fig. 3a,b). However SPs induced low-titers of anti-SPs IgG (Fig. 3c). In the case of group 4 (OVA + TMV) the anti-TMV titers did not statistically differ from the titers of mice sera in group 1 (PBS) (Fig. 3d). The initial concentration of all viruses on the microplate was 10 μg/ml, however, the IgG level to CaMV was very high, which caused problems with titer definition. Therefore, concentration of this virus was adjusted to 1 μg/ml. Notably, in spite of the lower concentration on the plate, titers to CaMV were the highest in this experiment. IgG titers to corresponding virus from each mouse are presented in Supplementary Table S5.

### Potentiation of immune response to the HEL

**Figure 4:**
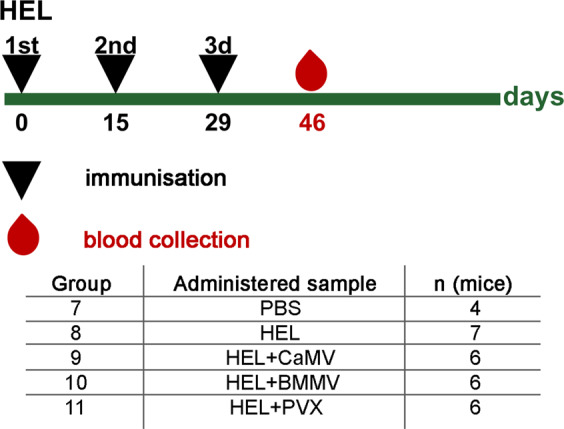
Immunisation schedule and description of animal groups in experiment with HEL. CD1 mice were immunised s.c. either with 5 μg of HEL or with 5 μg of HEL in composition of 100 μg of plant virus-based adjuvant (CaMV/BMMV/PVX). The control group was immunised with PBS. All administered samples were in PBS in total volume 0.2 ml. HEL, hen egg lysozyme; CaMV, Cauliflower mosaic virus; BMMV, bean mild mosaic virus; PVX, Potato virus X; n, number of animals.

**Figure 5:**
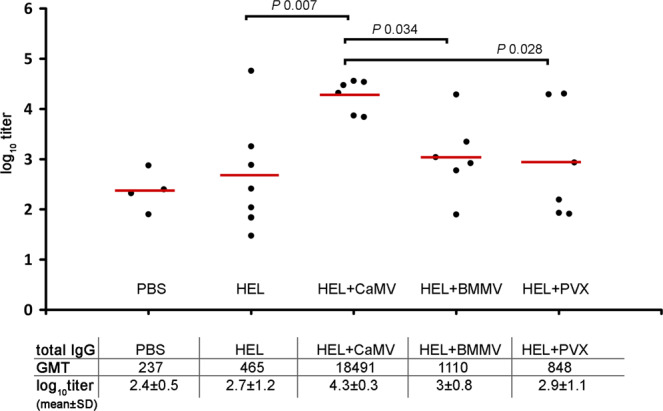
Analysis of IgG titers to HEL. Groups of mice were immunised s.c. three times (days 0, 15, 29). Blood was collected after the third immunisation on the 46^th^ day of the experiment. Sera titers were evaluated using an indirect ELISA with anti-mouse HRP conjugate against IgG (ab6728, Abcam, Cambridge, MA, USA). Concentration of HEL on microplate – 150 μg/ml, ● – anti-HEL serum log_10_titer from each mouse,  – mean. *P* values were calculated using a post hoc Dunn’s multiple-comparison test, which was conducted after a Kruskal-Wallis test. Kruskal-Wallis test *P* value: 0.037. GMT, geometric mean titer, SD, standard deviation.

Adjuvants may have different immunostimulating effects on various antigens^17,41^. Therefore, for our next experiment we selected another one model antigen (HEL) with a lower molecular weight (14 kDa)^42^ and a higher pI (11.35)^43^. As the potential adjuvants, we used the following viruses: CaMV, which showed high immunostimulation efficiency with OVA; PVX, as we expected that it would reveal adjuvant potential with HEL; BMMV, a virus which has a similar shape and size to CaMV. However, unlike CaMV, the genome of BMMV is represented by RNA. The mice were inoculated at days 0, 15, 29 s.c. with PBS, HEL and HEL-plant virus compositions (Fig. 4). TEM micrographs of HEL-viruses compositions are presented in Supplementary Fig. S3. The BMMV mean diameter was 35.9 ± 2.6 nm (Supplementary Fig. S3d) and the diameter of CaMV and length of PVX are indicated in Section 1 of the “Results”. TEM analysis showed the potential HEL non-covalent binding with BMMV and PVX (Supplementary Fig. S3c, Fig S3e). As in the case with OVA, the visible interaction of CaMV with HEL was not detected (Supplementary Fig. S3b). Blood was collected on day 46. The indirect ELISA analysis of mice sera was performed to determine the immune response (Fig. 5; Supplementary Table S6.). CaMV, as in the case of the first model antigen (OVA), effectively enhanced anti-HEL titers as compared with HEL alone (group 8). Interestingly, whilst having a similar shape and size to CaMV, BMMV had a significantly lower adjuvant potential (*P* value 0.034). PVX again did not exhibit any effective adjuvant properties.

### Immune response to the plant viruses in composition with HEL

**Figure 6:**
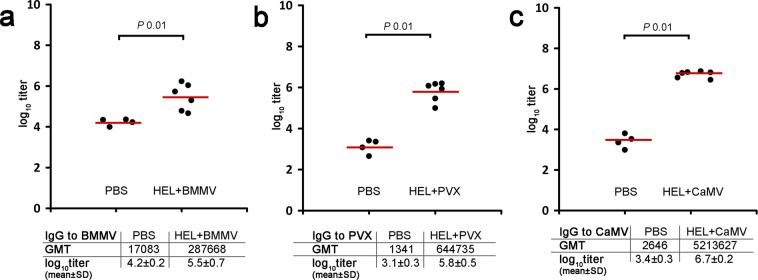
Analysis of total IgG titers to plant viruses after immunisation with HEL-plant virus compositions. (**a**) Total IgG titers to BMMV; (**b)** total IgG titers to PVX; (**c**) total IgG titers to CaMV. Groups of mice were immunised s.c. three times (days 0, 15, 29). Blood was collected after the third immunisation on the 46^th^ day of the experiment. Sera titers were evaluated using an indirect ELISA with anti-mouse HRP conjugate against IgG (ab6728, Abcam, Cambridge, MA, USA). Concentration of BMMV and PVX on microplate – 10 μg/ml of CaMV – 1 μg/ml, ● – serum log_10_titer from each mouse,  – mean. A Wilcoxon-Mann-Whitney two-tailed test was used to compare the statistical difference between groups. GMT, geometric mean titer, SD, standard deviation.

Regardless of the effectiveness of anti-HEL IgG stimulation, sera from groups 9, 10 and 11 were analysed for the presence of plant virus self-IgG. In this experiment, all viruses elicited a relatively high self-immunity after immunisations in composition with HEL: IgG titers to BMMV – 287668 (Fig. 6a); IgG titers to PVX – 644735 (Fig. 6b); IgG titers to CaMV –5213627 (Fig. 6c). However, CaMV induced the highest IgG titers to itself, as well as in an experiment with OVA. IgG titers to corresponding virus from each mouse are presented in Supplementary Table S7.

## Discussion

Vaccines are powerful tools to control and prevent infectious diseases in humans and animals^44,45^. The necessity to have safer and more effective vaccines drives the development of novel non-replicating forms. However, this type of vaccines often has an insufficient immunogenicity and requires the addition of various adjuvants, which play an essential role in improving the immune response to vaccine components^46^.

Aluminum compounds are the most widely used adjuvants in human vaccines^47^. However, these adjuvants are relatively weak in the antibody induction to certain antigens and in the induction of the Th1-immune response necessary to combat intracellular pathogens. Aluminum adjuvants have been reported to affect the physical and chemical stability of vaccine antigens over time and even to accelerate protein degradation^48,49^. Furthermore, aluminum salts are able to decrease the immunogenicity of vaccines but increase local reactogenicity^50^. In this regard, the search for both safe and effective adjuvants is a pressing issue worldwide.

Plant viruses, their VLP and structurally modified virus particles are promising candidates for the development of new generation adjuvants^1,6,44,51,52^. The adjuvant properties after simple mixing with antigens have already been shown for papaya mosaic virus virions and VLP, and for the structurally modified spherical particles of tobacco mosaic virus (SPs)^17–21^. Here we report on the adjuvant potential of plant viruses of various shapes, sizes and structure using OVA and HEL as the model antigens.

In our first experiment, we used rod-shaped TMV (*Tobamovirus*, *Virgaviridae*), filamentous PVX (*Potexvirus*, *Alfaflexiviridae*) and spherical CaMV (*Caulimovirus*, *Caulimaviridae*) as potential adjuvants and OVA as a model antigen. SPs were applied as a platform and potential adjuvant, whose immunostimulating properties have been shown previously^18,20,21,31^. In our preceding works we have described the adjuvant properties for SPs of size 100, 300 and 500 nm^18,20,21^. We have not revealed any distinctions in the immunostimulating properties of SPs of different sizes^18^. Here, we have used SPs with a mean diameter of 611 nm. It is important to emphasise that TMV and PVX are widely used to obtain chimeric virus particles (ChVP) displaying the epitopes of different pathogens^6,53^. For TMV and PVX ChVP, the immunostimulating effect has been demonstrated on fused epitopes^54–58^ even without any additional adjuvants^55,59,60^. Athough OVA is a relatively weak immunogen, here, we have demonstrated TMV, CaMV and SPs increasing anti-OVA IgG titers by nine, 13 and 15 times respectively, in comparison with the OVA alone. Unexpectedly, no differences in IgG titers between mice immunised with individual OVA and a composition of OVA + PVX were revealed. Thus, in addition to SPs, whose ability of which to improve immune response to non-covalently bound antigens has already been described, an effective adjuvant potential was shown for CaMV and TMV simply mixed with OVA.

Virus and protein-based carriers/adjuvants are known to be able to activate a self-immune response^35,54,61^. Some carriers (for example poxviruses and adenoviruses) can suffer from self-antibodies, in particular from pre-existing antibodies which can cause a clearance of virus/protein-based carriers by neutralising antibodies^62–64^. However, it has been shown for chimeric TMV, PapMV, cowpea mosaic virus and flagellin, that the existence of specific antibodies does not interfere with their immunostimulating properties^5,35,59,61,65^. Thereby, we found it necessary to evaluate IgG titers to adjuvants we examined. In the sera from group 3 (OVA + SPs), IgG titers to SPs were low, but showed a statistically significant difference with titers in the group immunised with PBS (*P* value 0.048). Probably, the low titers to SPs associated with the formation of SPs complexes with OVA (Supplementary Fig. S1b) and the masking of SPs epitopes from the immune system, thus, predominantly produced anti-OVA IgG. These data on anti-IgG SPs are consistent with our previous work, in which we showed that complexes of SPs with recombinant rubella antigen induced low anti-SPs titers^21^. In the sera from group 4 (OVA + TMV), with high IgG titers to OVA, anti-TMV IgG were considerably low and did not differ statistically from the sera titers of group 1 (PBS). Interestingly, in contrast to TMV, another helical plant virus, PVX, induced a relatively high self-immune response in the absence of effective stimulation of IgG to OVA. In group 5 (OVA + CaMV), there was a fairly strong humoral response against CaMV. Thus, we did not identify any linkage between the effectiveness of the immune response to a model antigen and self-immunity. However, CaMV was the only virus for which we did not detect any visible interaction with OVA (Supplementary Fig. S1f). Potentially, such a difference may contribute to considerably high levels of anti-CaMV IgG in comparison with titers to other plant virus-based adjuvants. The high self-IgG titers to CaMV, in spite of a relatively efficient immune response to OVA, aroused our interest and we decided to analyse the adjuvant potential of this virus with another model antigen.

Adjuvants and plant viruses, in particular, are known to stimulate immune response to various antigens with different efficiency^17^. Therefore, for our subsequent experiment, we tried another model antigen – HEL. Nevertheless, PVX in mixture with OVA did not prove to be an effective adjuvant. We had hoped that with HEL, it would manifest immunostimulating potential as had been shown for another potexvirus with a similar structure, shape and size (papaya mosaic virus)^17^. In our experiment with HEL, CaMV was selected as a potentially effective adjuvant. We also chose another icosahedral virus, bean mild mosaic virus (BMMV, *Tombusviridae*), which has a similar size of virions to CaMV, but different genomic nucleic acid (Table 1). CaMV, as with OVA, demonstrated good adjuvant properties on HEL. In group 9 (HEL + CaMV) the IgG titers to HEL increased 40 times in comparison with group 8 immunised with HEL alone, and 17 times in comparison with group 10 (HEL + BMMV). Possibly, such a contrast in the adjuvant properties of two viruses that are similar in shape and size may be related to difference in genomic nucleic acids: CaMV is a DNA virus, while BMMV is an RNA virus. This suggestion is in line with the information that DNA itself may has a stronger adjuvant effect than RNA^66^. Also may be important structural differences between virions of CaMV (420 protein subunits per capsid, T = 7) and BMMV (180 protein subunits per capsid, T = 3; data for the family *Tombusviridae* according ViralZone)^67,68^. For group 11, being immunised with HEL + PVX no significant differences in sera IgG titers were detected in comparison with group 8, which was immunised by individual HEL. In comparison with CaMV, PVX induced 22 times lower anti-HEL IgG titers. In this experiment, all viruses induced a relatively high self-immunity: TEM allows to suppose, that the considerably high immune response to CaMV was attributable in part to the absence of observable binding between HEL and CaMV (Supplementary Fig S3b). According to the results of the experiment with HEL, the shape and size of virions, apparently, do not play a key role in immunostimulating properties.

**Table 1:** Summary data for the adjuvant properties of plant virus particles with various shapes and sizes.

Potential adjuvant	Shape/size (nm)		Nucleic acid	Immunostimulation of IgG to a model antigen	Self-IgG
SPs		611 ± 71	No	high	low
TMV		315 ± 27 (l)	RNA	high	ns*
PVX		521 ± 39 (l)	RNA	ns*	high
CaMV		35 ± 2; T = 7	DNA	high	high
BMMV		36 ± 3; T = 3	RNA	ns*	high

The table contains a description of the viral particles tested, as well as the relative level of immune response to each component of the compositions administered to mice. *ns – not significant, l – length, T – triangulation number. T numbers are indicated according^67,68^. TMV, Tobacco mosaic virus; CaMV, Cauliflower mosaic virus; PVX, Potato virus X; SPs, spherical particles obtained by thermal remodelling of TMV; BMMV, bean mild mosaic virus.The virions and SPs images are not drawn to scale.

Hence, we can conclude that CaMV and TMV are able to efficiently stimulate an immune response to the model antigen (Table 1), as has been shown previously for SPs^18,20,21^. However, CaMV induces rather a strong self-immune response, which may cause concern and requires further analysis of potential systemic toxicity risks. Nonetheless, to our knowledge, this is the first report describing the adjuvant properties of a DNA-containing plant virus, which can effectively stimulate not only total IgG titers, but also IgG1, IgG2a and IgG2b isotypes (Fig. 2, Fig. 5 and Supplementary Fig. S2). Probably, the DNA-genome of CaMV or capsid structural features play a role both in stimulating the immune response to the target antigen, and in stimulating self-immunity. Further research is required to identify the key participants in the stimulation of the immune response by CaMV. It is interesting that RNA-free SPs can stimulate comparable total IgG titers as the viruses containing RNA (TMV) or DNA (CaMV). In addition, we did not reveal any differences in the stimulation of total IgG by TMV and CaMV. The low self-IgG titers to TMV and SPs enable an assumption that these adjuvants might be useful, even for pre-exposed individuals. It should also be emphasised that procedures for obtaining TMV and SPs are not expensive, and can be easily scaled for different biotechnological purposes. Remarkably, SPs are RNA-free protein structures, which makes their use safe not only for humans and mammals, but also for the environment.

On the whole, our data did not reveal any clear influence of plant viruses’ size or shape on the humoral immune response to the model antigen as well as the presence or absence of RNA. Given the immunostimulating properties to the model antigen and self-immunity to potential adjuvants (Table 1), in our opinion, the most interesting objects are SPs and TMV. The data obtained indicate that adjuvants based on plant viruses can be considered to be a promising tool in further vaccine development.

## Methods

### Viruses and SPs production

TMV, PVX, CaMV and BMMV were propagated and purified as described previously^24,25,69–71^. SPs were obtained, as described earlier, from TMV with a concentration of 2 mg/ml^22,24^.

### Transmission electron microscopy (TEM)

The samples were obtained and analysed, as described previously, by TEM microscopes JEOL JEM-1400, JEM-1400Flash and JEM-1011(JEOl, Japan), and 2% uranyl acetate was used as a contrast agent. Samples of free SPs did not contrast^71^. The scientific image manipulation software ImageJ (National Institutes of Health, USA) was used for size calculations.

### Immunisations

All mice experiments were conducted in accordance with experimental practices and standards accepted by the Ethics Committee of the Institute of Gene Biology, Russian Academy of Sciences. Groups of CD1 mice (n = 6) were immunised s.c. with either 5 μg of OVA (Sigma, A5503-1G) or 5 μg of OVA with 100 μg of SPs/TMV/PVX/СaMV in a total volume of 0.2 ml. The control group of CD1 mice (n = 5) was immunised with PBS. In a separate experiment, groups of CD1 mice (n = 4–7) were immunised s.c. with either PBS, 5 μg of HEL (Roche, 10837059001) or 5 μg of HEL with 100 μg of СaMV/BMMV/PVX in a total volume of 0.2 ml. The schedule of immunisation and blood collection is presented in the Fig. 1 and Fig. 4. The research was approved by the Ethics Committee of the Institute of Gene Biology, Russian Academy of Sciences.

### ELISA

Microplates (Greiner Bio-One 655001, Germany) were coated with 10 μg/mL of OVA/ SPs/TMV/PVX/BMMV, 150 μg/mL of HEL or 1 μg/mL of CaMV and then blocked with a PBS containing 1% skimmed milk. Murine sera were diluted in PBS with 1% skimmed milk and titrated with 1:3 dilutions. Anti-mouse HRP conjugates (Abcam, Cambridge, MA, USA) against IgG (ab6728), IgG1(ab97240), IgG2a (ab97245) and IgG2b (ab97250) were used in a dilution of 1:10000 and anti-mouse HRP conjugate against IgG3 (ab97260) was used in a dilution of 1:5000. Staining was performed using 3,3′,5,5′-tetramethylbenzidine substrate (PanReac AppliChem, A3840,0001). The reaction was quenched by the addition of 2 M H_2_SO_4_, OD measured at 450 nm. A positive titer was defined as three standard deviations above the mean value of the block. The geometric mean titers were calculated for each group.

### Statistical analysis

For multiple comparisons, the Kruskal-Wallis test and a post hoc Dunn’s test were used. The Exact Wilcoxon-Mann-Whitney Test Calculator was used to compare differences between two groups and when comparing the non-adjuvanted group with the other groups^72^. The statistical tests used in each individual experiment have been indicated in the captions to the figures. Probability values (*P* values) of less than 0.05 were considered to be significant.

## Supplementary information

Supplementary Information.

## Data Availability

All the relevant data are provided in this paper and in Supporting Information files.
