# Supplementary material for: Plant virus particles with various shapes as potential adjuvants
Source: Sci Rep. 2020 Jun 25;10:10365. doi: 10.1038/s41598-020-67023-4 (PMC7316779; doi:10.1038/s41598-020-67023-4)
Supplement: Supplementary file 1 — Supplementary Information. [file 41598_2020_67023_MOESM1_ESM.pdf]

Plant virus particles with various shapes as potential adjuvants

Ekaterina A. Evtushenko[1]*, Ekaterina M. Ryabchevskaya[1], Nikolai A. Nikitin[1], Joseph G. Atabekov[1] & Olga V. Karpova[1]

[1]Department of Virology, Faculty of Biology, Lomonosov Moscow State University, 1-12 Leninskie Gory, Moscow, 119234, Russian Federation.

***Corresponding author:** Ekaterina A. Evtushenko, **trifonova.katerina@gmail.com**

Supplementary information

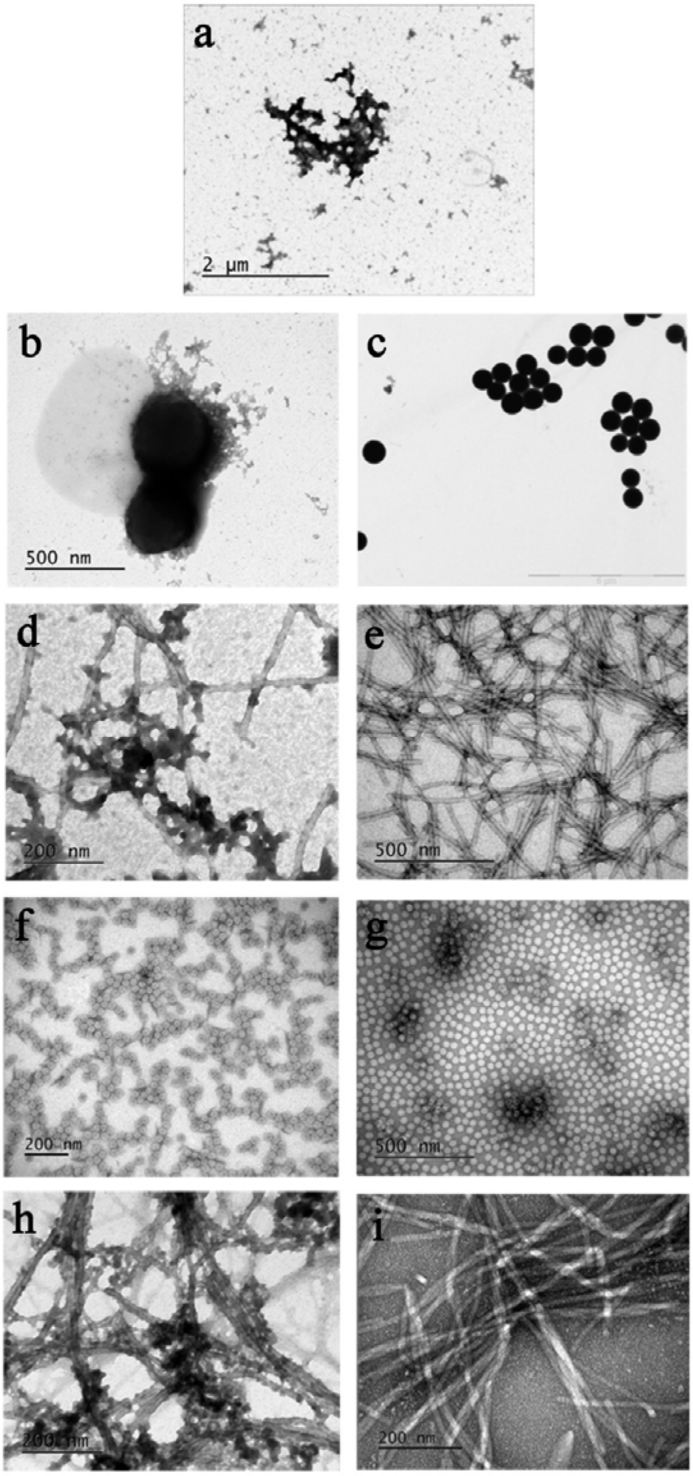

Fig. S1 Characterisation of OVA-virus/SPs compositions for immunisation. a, OVA; **b,** OVA with SPs; **d,** OVA with TMV; **f,** OVA with CaMV; **h**, OVA with PVX; **c, e, g, i:** SPs, TMV, CaMV, PVX (controls without OVA). The concentration of OVA (0.025 mg/ml) and virions/SPs (0.5 mg/ml) was the same as for the samples for immunisation. All samples were in PBS. Transmission electron microscopy, a, b, d, e, f, g, h, i: staining with 2% uranyl acetate; c, without staining. Scale bars: c, 5 µm; h, 200 nm.

a

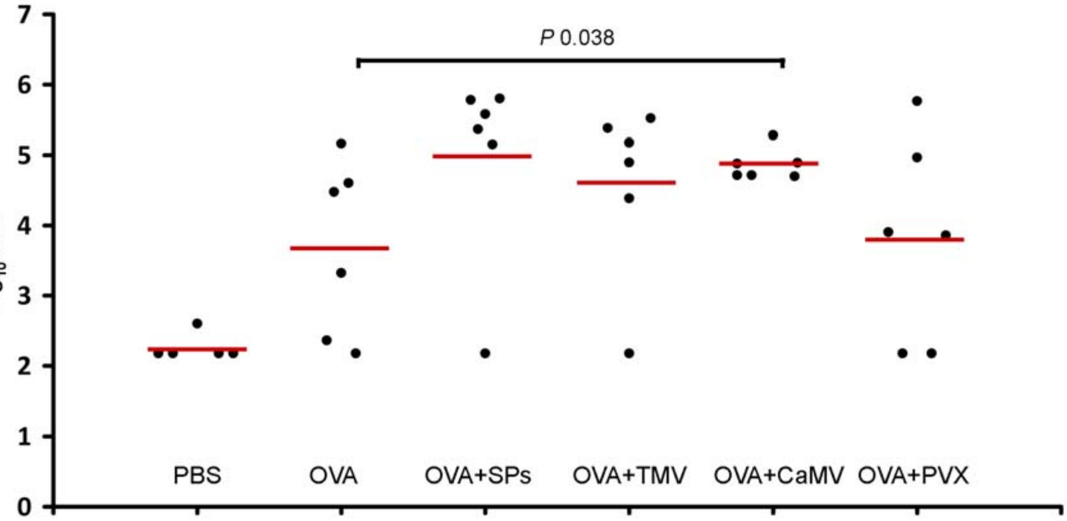

IgG1	PBS	OVA	OVA+SPs	OVA+TMV	OVA+CaMV	OVA+PVX
GMT	183	4843	94397	38844	73076	6418
log_{10}titer (mean±SD)	2.3±0.2	3.7±1.3	5.0±1.4	4.6±1.3	4.9±0.2	3.8±1.5

b

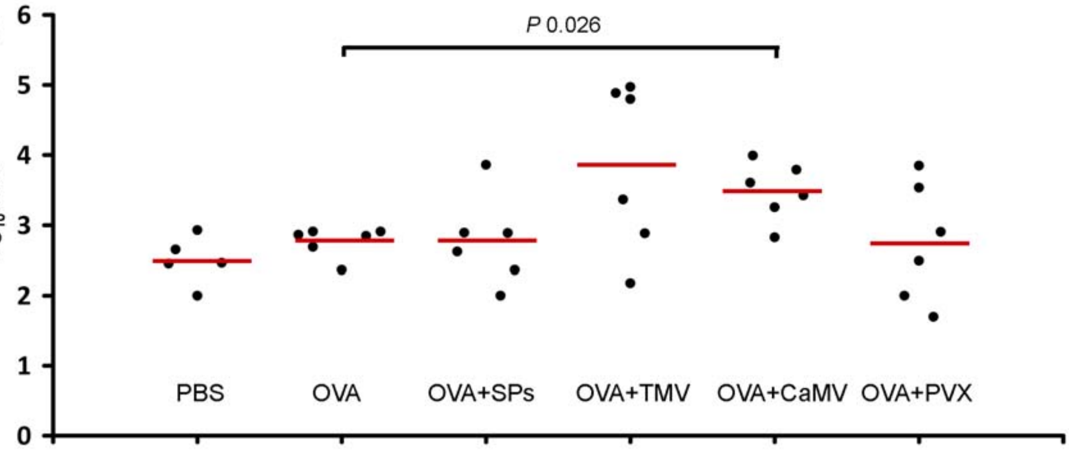

IgG2a	PBS	OVA	OVA+SPs	OVA+TMV	OVA+CaMV	OVA+PVX
GMT	315	578	590	7024	3032	557
log_{10}titer (mean±SD)	2.5±0.3	2.8±0.2	2.8±0.6	3.8±1.2	3.5±0.4	2.7±0.8

c

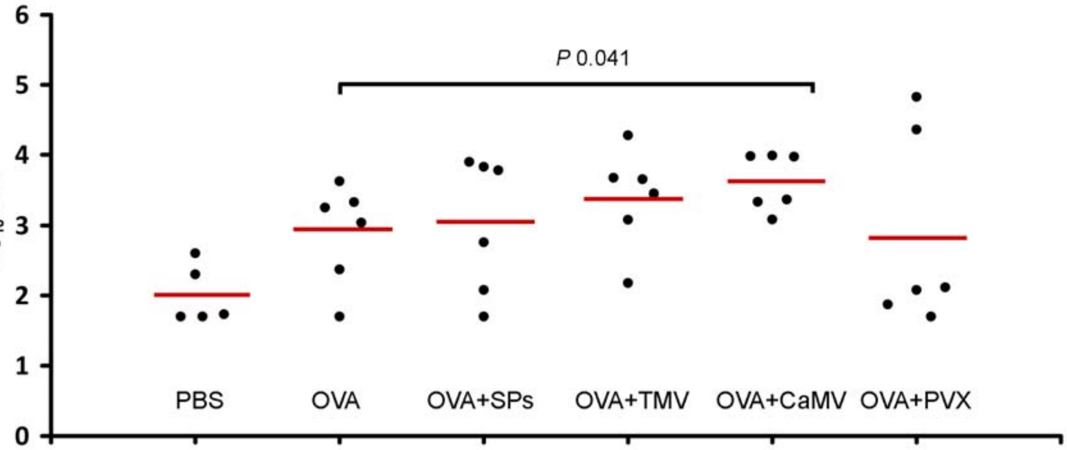

IgG2b	PBS	OVA	OVA+SPs	OVA+TMV	OVA+CaMV	OVA+PVX
GMT	102	772	1015	2422	4199	668
log_{10}titer (mean±SD)	2.0±0.4	2.9±0.7	3.0±1.0	3.4±0.7	3.6±0.4	2.8±1.4

Fig. S2. Analysis of IgG1, IgG2a and IgG2b titers to OVA. a, IgG1 titers, **b**, IgG2a titers, **c,** IgG2b titers. Groups of mice were immunised s.c. four times (days 0, 15, 29, 43). Blood was collected after the fourth immunisation on the 56th day of the experiment. Sera titers were evaluated using an indirect ELISA with Abcam anti-mouse HRP conjugates (Cambridge, MA, USA) against: a, IgG1(ab97240); b, IgG2a (ab97245); c, IgG2b (ab97250). Concentration of OVA on microplate – 10µg/ml, ● – – anti-OVA serum $\log_{10}$titer from each mouse, ▬ – mean. A Wilcoxon-Mann-Whitney two-tailed test was used to compare the statistical differences between groups 3,4,5,6 and group 2, which had no adjuvant. Data on the graphs are expressed as $\log_{10}$. GMT – geometric mean titer, SD – standard deviation.

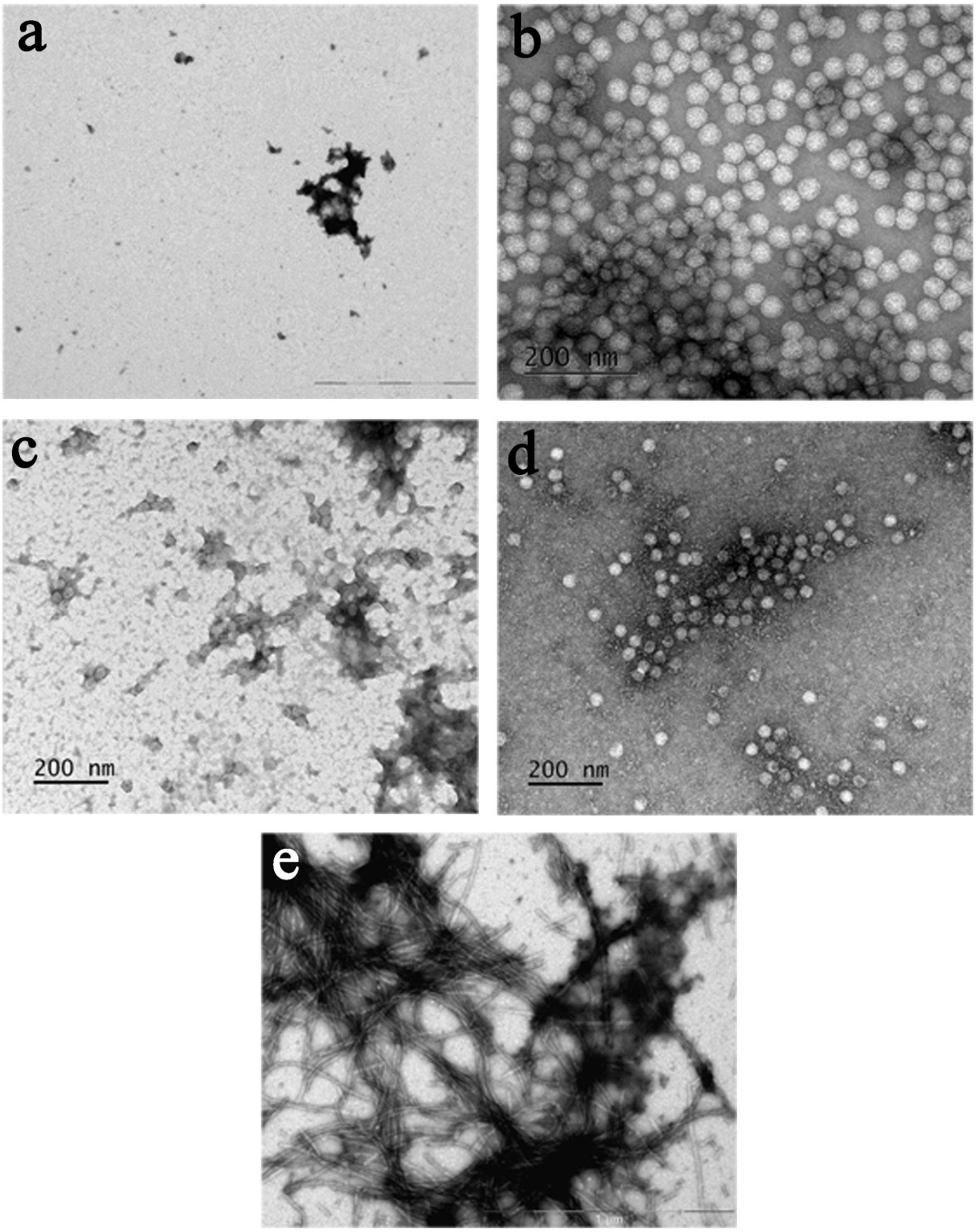

Fig. S.3 Characterisation of HEL-virus compositions for immunisation. a, HEL; **b,** HEL with CaMV; **c**, HEL with BMMV; **d,** BMMV (control without HEL); **e,** HEL with PVX. The concentration of HEL (0.025 mg/ml) and virions (0.5 mg/ml) was the same as for the samples for immunisation. All samples were in PBS. Transmission electron microscopy, staining with 2% uranyl acetate. Scale bars: a, e, 1 µm.

total IgG to OVA			
**group**	**identification number of mouse**	**titer**	**log_{10} titer**
group 1 (PBS)	1	2900	3.462
	2	3100	3.491
	3	3120	3.494
	4	877	2.943
	5	3150	3.498
group 2 (OVA)	6	796	2.901
	7	1250	3.097
	8	49400	4.694
	9	954	2.98
	10	9510	3.978
	11	2100	3.322
group 3 (OVA + SPs)	12	190000	5.279
	13	191000	5.281
	14	2430	3.386
	15	39000	4.591
	16	52200	4.718
	17	68300	4.834
group 4 (OVA + TMV)	18	60500	4.782
	19	120000	5.079
	20	63000	4.799
	21	1300	3.114
	22	27000	4.431
	23	24300	4.386
group 5 (OVA + CaMV)	24	22800	4.358
	25	69000	4.839
	26	26000	4.415
	27	29400	4.468
	28	64300	4.808
	29	60300	4.78
group 6(OVA + PVX)	30	230000	5.362
	31	1150	3.061
	32	1000	3
	33	2400	3.38
	34	59000	4.771
	35	2300	3.362

Tab. S1 Total IgG titers OVA.

Groups of CD1 mice were immunised s.c. four times (days 0, 15, 29, 43) either with 5 µg of OVA or with 5 µg of OVA in composition of 100 µg of plant virus-based adjuvant (SPs/TMV/PVX/CaMV). The control group was immunised with PBS. All administered samples were in PBS in total volume 0.2 ml.
Blood was collected after the fourth immunisation on the 56th day of the experiment. Sera titers were evaluated using an indirect ELISA with anti-mouse HRP conjugate against IgG (ab6728, Abcam, Cambridge, MA, USA). Concentration of OVA on microplate – 10µg/ml.

IgG1 to OVA			
**group**	**identification number of mouse**	**titer**	**log_{10} titer**
group 1 (PBS)	1	400	2.602
	2	150	2.176
	3	150	2.176
	4	150	2.176
	5	150	2.176
group 2 (OVA)	6	230	2.362
	7	2100	3.322
	8	147000	5.167
	9	150	2.176
	10	40400	4.606
	11	30000	4.477
group 3 (OVA + SPs)	12	600000	5.778
	13	377000	5.576
	14	150	2.176
	15	230000	5.362
	16	634000	5.802
	17	143000	5.155
group 4 (OVA + TMV)	18	240000	5.38
	19	330000	5.519
	20	24400	4.387
	21	150	2.176
	22	150000	5.176
	23	79000	4.898
group 5 (OVA + CaMV)	24	50000	4.699
	25	76000	4.881
	26	52000	4.716
	27	52000	4.716
	28	190000	5.279
	29	78000	4.892
group 6(OVA + PVX)	30	580000	5.763
	31	150	2.176
	32	150	2.176
	33	8000	3.903
	34	93000	4.968
	35	7200	3.857

Tab. S2 IgG1 titers OVA.

Groups of CD1 mice were immunised s.c. four times (days 0, 15, 29, 43) either with 5 µg of OVA or with 5 µg of OVA in composition of 100 µg of plant virus-based adjuvant (SPs/TMV/PVX/CaMV). The control group was immunised with PBS. All administered samples were in PBS in total volume 0.2 ml.
Blood was collected after the fourth immunisation on the 56th day of the experiment. Sera titers were evaluated using an indirect ELISA with anti-mouse HRP conjugate against against IgG1 (ab97240, Abcam, Cambridge, MA, USA). Concentration of OVA on microplate – 10µg/ml.

IgG2a to OVA			
**group**	**identification number of mouse**	**titer**	**log_{10} titer**
group 1 (PBS)	1	280	2.447
	2	450	2.653
	3	850	2.929
	4	100	2
	5	290	2.462
group 2 (OVA)	6	490	2.69
	7	810	2.908
	8	724	2.86
	9	230	2.362
	10	700	2.845
	11	805	2.906
group 3 (OVA + SPs)	12	780	2.892
	13	100	2
	14	420	2.623
	15	767	2.885
	16	230	2.362
	17	7280	3.862
group 4 (OVA + TMV)	18	76500	4.884
	19	93700	4.972
	20	2330	3.367
	21	760	2.881
	22	63100	4.8
	23	150	2.176
group 5 (OVA + CaMV)	24	1790	3.253
	25	9830	3.993
	26	2650	3.423
	27	6200	3.792
	28	4010	3.603
	29	670	2.826
group 6(OVA + PVX)	30	3430	3.535
	31	310	2.491
	32	50	1.699
	33	100	2
	34	7030	3.847
	35	802	2.904

Tab. S3 IgG2a titers OVA.

Groups of CD1 mice were immunised s.c. four times (days 0, 15, 29, 43) either with 5 µg of OVA or with 5 µg of OVA in composition of 100 µg of plant virus-based adjuvant (SPs/TMV/PVX/CaMV). The control group was immunised with PBS. All administered samples were in PBS in total volume 0.2 ml. Blood was collected after the fourth immunisation on the 56th day of the experiment. Sera titers were evaluated using an indirect ELISA with anti-mouse HRP conjugate against IgG2a (ab97245, Abcam, Cambridge, MA, USA). Concentration of OVA on microplate – 10µg/ml.

IgG2b to OVA			
**group**	**identification number of mouse**	**titer**	**log_{10} titer**
group 1 (PBS)	1	400	2.602
	2	200	2.301
	3	50	1.699
	4	50	1.699
	5	54	1.732
group 2 (OVA)	6	1100	3.041
	7	1800	3.255
	8	2160	3.334
	9	50	1.699
	10	235	2.371
	11	4200	3.623
group 3 (OVA + SPs)	12	7900	3.898
	13	6000	3.778
	14	50	1.699
	15	574	2.759
	16	120	2.079
	17	6700	3.826
group 4 (OVA + TMV)	18	4700	3.672
	19	4490	3.652
	20	1200	3.079
	21	150	2.176
	22	2800	3.447
	23	19000	4.279
group 5 (OVA + CaMV)	24	2180	3.338
	25	9600	3.982
	26	1220	3.086
	27	9760	3.989
	28	9400	3.973
	29	2340	3.369
group 6(OVA + PVX)	30	23000	4.362
	31	130	2.114
	32	74	1.869
	33	120	2.079
	34	67000	4.826
	35	50	1.699

Tab. S4 IgG2b titers OVA.

Groups of CD1 mice were immunised s.c. four times (days 0, 15, 29, 43) either with 5 µg of OVA or with 5 µg of OVA in composition of 100 µg of plant virus-based adjuvant (SPs/TMV/PVX/CaMV). The control group was immunised with PBS. All administered samples were in PBS in total volume 0.2 ml. Blood was collected after the fourth immunisation on the 56th day of the experiment. Sera titers were evaluated using an indirect ELISA with anti-mouse HRP conjugate against IgG2b (ab97250, Abcam, Cambridge, MA, USA). Concentration of OVA on microplate – 10µg/ml.

total IgG to potential adjuvants. experiment with OVA			
**group**	**identification number of mouse**	**titer**	**log_{10} titer**
group 1 (PBS) titrated on SPs	1	790	2.898
	2	120	2.079
	3	240	2.38
	4	120	2.079
	5	290	2.462
group 3 (OVA + SPs) titrated on SPs	12	3200	3.505
	13	1400	3.146
	14	1200	3.079
	15	2700	3.431
	16	130	2.114
	17	639	2.806
group 1 (PBS) titrated on TMV	1	730	2.863
	2	150	2.176
	3	420	2.623
	4	120	2.079
	5	3300	3.519
group 4 (OVA + TMV) titrated on TMV	18	936	2.971
	19	257	2.41
	20	360	2.556
	21	310	2.491
	22	12000	4.079
	23	9300	3.968
group 1 (PBS) titrated on CaMV	1	150	2.176
	2	140	2.146
	3	550	2.74
	4	350	2.544
	5	260	2.415
group 5 (OVA + CaMV) titrated on CaMV	24	25000000	7.398
	25	41000000	7.613
	26	9190000	6.963
	27	14500000	7.161
	28	8200000	6.914
	29	66900000	7.825
group 1 (PBS) titrated on PVX	1	7800	3.892
	2	400	2.602
	3	380	2.58
	4	160	2.204
	5	380	2.58
group 6 (OVA + PVX) titrated on PVX	30	1500000	6.176
	31	640000	5.806
	32	6560000	6.817
	33	2200000	6.342
	34	5100000	6.708
	35	6500000	6.813

Tab. S5 Total IgG titers to potential adjuvants (SPs, TMV, CaMV, PVX) after immunisation with OVA-plant virus particles compositions.

Groups of CD1 mice were immunised s.c. four times (days 0, 15, 29, 43). Blood was collected after the fourth immunisation on the 56th day of the experiment. Sera titers were evaluated using an indirect ELISA with anti-mouse HRP conjugate against IgG (ab6728, Abcam, Cambridge, MA, USA).Concentration of TMV, SPs, PVX on microplate – 10µg/ml of CaMV – 1 µg/ml.

total IgG to HEL			
**group**	**identification number of mouse**	**titer**	**log_{10} titer**
group 7 (PBS)	36	210	2.322
	37	750	2.875
	38	80	1.903
	39	250	2.398
group 8(HEL)	40	30	1.477
	41	260	2.415
	42	110	2.041
	43	69	1.839
	44	57600	4.76
	45	770	2.886
	46	1800	3.255
group 9 (HEL + CaMV)	47	7390	3.869
	48	34400	4.537
	49	36200	4.559
	50	29900	4.476
	51	20900	4.32
	52	6950	3.842
group 10 (HEL + BMMV)	53	79	1.898
	54	600	2.778
	55	1100	3.041
	56	19400	4.288
	57	2230	3.348
	58	830	2.919
group 11 (HEL + PVX)	59	85	1.929
	60	859	2.934
	61	157	2.196
	62	20200	4.305
	63	19600	4.292
	64	82	1.914

Tab. S6 Total IgG titers HEL.

Groups of CD1 mice were immunised s.c. three times (days 0, 15, 29) either with 5 µg of HEL or with 5 µg of HEL in composition of 100 µg of plant virus-based adjuvant (CaMV/BMMV/PVX). The control group was immunised with PBS. All administered samples were in PBS in total volume 0.2 ml. Blood was collected after the third immunisation on the 46th day of the experiment. Sera titers were evaluated using an indirect ELISA with anti-mouse HRP conjugate against IgG (ab6728, Abcam, Cambridge, MA, USA). Concentration of HEL on microplate – 150µg/ml.

total IgG to potential adjuvants. experiment with HEL			
**group**	**identification number of mouse**	**titer**	**log_{10} titer**
group 7 (PBS) titrated on CaMV	36	2300	3.362
	37	6400	3.806
	38	980	2.991
	39	3400	3.531
group 9 (HEL + CaMV) titrated on CaMV	47	6100000	6.785
	48	3600000	6.556
	49	6500000	6.813
	50	6700000	6.826
	51	7500000	6.875
	52	2800000	6.447
group 7 (PBS) titrated on BMMV	36	22000	4.342
	37	17000	4.23
	38	9900	3.996
	39	23000	4.362
group 10 (HEL + BMMV) titrated on BMMV	53	61000	4.785
	54	200000	5.301
	55	46000	4.663
	56	1700000	6.23
	57	1100000	6.041
	58	540000	5.732
group 7 (PBS) titrated on PVX	36	2600	3.415
	37	2300	3.362
	38	450	2.653
	39	1200	3.079
group 11 (HEL + PVX) titrated on PVX	59	100000	5
	60	1200000	6.079
	61	860000	5.934
	62	1500000	6.176
	63	290000	5.462
	64	1600000	6.204

Tab. S7 Total IgG titers to potential adjuvants (CaMV, BMMV, PVX) after immunisation with HEL-plant virus particles compositions.

Groups of CD1 mice were immunised s.c. three times (days 0, 15, 29). Blood was collected after the third immunisation on the 46th day of the experiment. Sera titers were evaluated using an indirect ELISA with anti-mouse HRP conjugate against IgG (ab6728, Abcam, Cambridge, MA, USA). Concentration of TMV, SPs, PVX on microplate – 10μg/ml of CaMV – 1 μg/ml.
